# Mucocèle appendiculaire secondaire à une tumeur coecale

**DOI:** 10.11604/pamj.2014.17.18.3635

**Published:** 2014-01-17

**Authors:** Hossam Behammane, Younes Aggouri

**Affiliations:** 1 Faculté de Médecine et de Pharmacie de Fès, Université Sidi Mohammed Ben Abdellah, Département de Chirurgie, CHU Hassan II Fès, Maroc

**Keywords:** Mucocèle appendiculaire, tumeur coecale, adénocarcinome, appendiceal mucocele, caecal tumor, adenocarcinoma

## Image en medicine

Le mucocèle appendiculaire est une lésion rare de l'appendice caractérisée par l'accumulation de mucus dans la lumière appendiculaire occasionnant une dilatation de la lumière appendiculaire. Sa pathogénie est controversée, deux théories physiopathologiques ont été développées : une théorie obstructive et une théorie néoplasique. L'obstacle peut être: une tumeur endocrine appendiculaire, non sécrétante, adénome ou adénocarcinome du cæcum ou de l'appendice, endométriose, compression extrinsèque par des nodules de carcinose péritonéale, infections spécifiques : stercolithe, corps étrangers, volvulus appendiculaire ou plicature, diverticule appendiculaire. Nous rapportons l'observation d'un patient âgé de 55 ans qui a présenté depuis 08 mois un syndrome subocclusif à répétition spontanément résolutif associé à une douleur abdominale localisée au niveau de la fosse iliaque droite avec notion d'amaigrissement chiffré à 8 Kg. L'examen clinique a mis en évidence une sensibilité au niveau de fosse iliaque droite sans masse palpale. Une Coloscopie réalisée a mis en évidence un processus bourgeonnant coecal. L'examen anatomopathologique de la biopsie est revenu en faveur d'un adénocarcinome bien différencié et infiltrant. Le scanner thoraco-abdomino pelvien a révélé un épaississement irrégulier du coecum et de la dernière anse iléale associé à une formation kystique latéro coecale interne faisant suspecter un mucocele appendiculaire. Le patient a été opérer par laparotomie mediane avec découverte en per opératoire d'une masse coecale dure associée à un mucocèle appendiculaire d'où la réalisation d'une hémicolectomie droite avec anastomose iléo colique término terminale. L'examen anatomopathologique de la pièce opératoire a mis en évidence un adénocarcinome colloïde muqueux du coecum circonférentiel arrivant jusqu'à la sous séreuse. Les limites de résection distale et proximale ont été saines. Présence de 12 nodules tumoraux dans la sous séreuse, engainement perinerveux, et d'un mucocéle appendiculaire. Le curage ganglionnaire a comporté 10 ganglions non métastatiques. La tumeur a été classée pT3N1C. Les suites opératoires ont été simples. Le patient a été adressé en oncologie pour chimiothérapie adjuvante après réunion de concertation multidisciplinaire. Sur un recul de 09 mois, pas de récidive locorégionale ni de métastase à distance.

**Figure 1:**
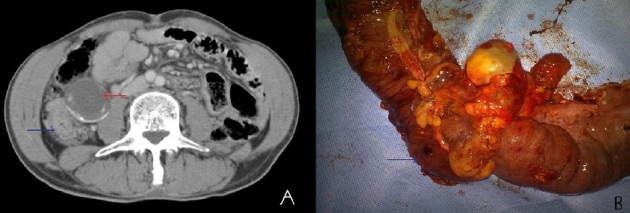
A) coupe scannographique abdominale montrant un épaississement irrégulier du coecum (flèche bleu) associé un mucocele appendiculaire (flèche rouge); B) Pièce opératoire montrant le mucocele appendiculaire (flèche rouge). Coecum (flèche bleu)

